# Hospital Readmission Prevalence and Analysis of Those Potentially Avoidable in Southern Italy

**DOI:** 10.1371/journal.pone.0048263

**Published:** 2012-11-02

**Authors:** Aida Bianco, Antonio Molè, Carmelo G. A. Nobile, Gabriella Di Giuseppe, Claudia Pileggi, Italo F. Angelillo

**Affiliations:** 1 Department of Health Sciences, Medical School, University of Catanzaro "Magna Græcia", Catanzaro, Italy; 2 Department of Experimental Medicine, Second University of Naples, Naples, Italy; Chancellor College, University of Malawi, Malawi

## Abstract

**Background:**

One quality indicator of hospital care, which can be used to judge the process of care, is the prevalence of hospital readmission because it reflects the impact of hospital care on the patient’s condition after discharge. The purposes of the study were to measure the prevalence of hospital readmissions, to identify possible factors that influence such readmission and to measure the prevalence of readmissions potentially avoidable in Italy.

**Methods:**

A sample of 2289 medical records of patients aged 18 and over admitted for medical or surgical illness at one 502-bed community non-teaching hospital were randomly selected.

**Results:**

A total of 2252 patients were included in the final analysis, equaling a response rate of 98.4%. The overall hospital readmission prevalence within 30 days of discharge was 10.2%. Multivariate logistic regression analysis revealed that the proportion of patients readmitted within 30 days of discharge significantly increased regardless of Charlson et al. comorbidity score, among unemployed or retired patients, and in patients in general surgery. A total of 43.7% hospital readmissions were judged to be potentially avoidable. Multivariate logistic regression analysis showed that potentially avoidable readmissions were significantly higher in general surgery, in patients referred to hospital by an emergency department physician, and in those with a shortened time between discharge and readmission.

**Conclusion:**

Additional research on intervention or bundle of interventions applicable to acute inpatient populations that aim to reduce potentially avoidable readmissions is strongly needed, and health care providers are urged to implement evidence-based programs for more cost-effective delivery of health care.

## Introduction

The appropriate hospital use is a growing concern in several countries since hospital care generates the largest amount of health expenditure. In this context, inappropriate or excessive access of hospitalization is a prominent issue, and the consistent measurement of hospital care processes represent a useful strategy to improve the quality and efficiency of health care delivered to patients [1]–[4].

Italy’s health care system is a regionally based National Health Service that provides universal coverage of the population free of charge at the point of service. Due to limited financial and specialized human resources, the publicly-funded and universal-access health care system is challenged by ever increasing demands. Effective control mechanisms must be implemented, and adequate efficient programs of health care must be delineated.

Over the past few years, many observational studies on large samples in the field of quality of hospital health care have been conducted. One quality indicator of hospital care, which can be used to judge the process of care, is the prevalence of hospital readmission because it reflects the impact of hospital care on the patient’s condition after discharge. Several studies have been made in various countries [5]–[13], but to our knowledge, no surveys have been published to date to assess the hospital readmission in Italy. Consequently, for the reasons stated above, the need of acquiring such information is increasingly perceived as a priority and, therefore, the purposes of the present study were to measure the prevalence of hospital readmissions, to identify possible factors that influence such readmission, and to measure the prevalence of readmissions potentially avoidable in Italy.

## Materials and Methods

This cross-sectional investigation was carried out from September 2005 through February 2007 at one 502-bed community non-teaching hospital located in the city of Catanzaro (Italy) which serves an area of approximately 370,000 inhabitants.

A sample of 2289 medical records of patients aged 18 and over admitted for medical or surgical illness were randomly selected. Each patient was approached to request participation by two trained physicians having experience in clinical documentation and who were not involved in care. Written informed consent was obtained from the subjects after explaining the study objectives. The subjects were free to withdraw at any time without giving any reason. Strict confidentiality was maintained throughout the process of data collection, entry, and analysis. For patients deemed too ill and unable to answer, the trained physicians attempted to contact a proxy. In this case, the data were collected with the informed consent of proxies. The trained physicians consulted with medical staff in the ward regarding each patient before making contact to request participation in the study. Patients were excluded if clinical staff deemed them medically or psychologically unable to provide informed consent to take part in the study or if patient behaviour was considered a threat to researcher safety at that time. The researcher recorded the reasons for exclusion.

The trained physicians collected the data by reviewing charts and by interviewing at bedside all patients on previous admission to the hospital. A hospital readmission was defined as a second consecutive admission occurred within 30 days to the same or another hospital in the country. Patients were ruled out if they left the hospital against medical advice during the first hospitalization.

Each readmission was classified as potentially avoidable or unavoidable by taking into account diagnostic and therapeutic process of the first admission. A researcher and an experienced physician concurrently used a set of criteria derived from previously published research (Table 1) [7], in order to assign the readmission into one of the two categories. An agreement was reached by a discussion between the two professionals in the case of discordant opinions.

**Table 1:** Causes of readmissions according to their potential avoidability.

*Potentially avoidable causes*
Complication of surgical procedure, except healthcare-related infection
Procedure not performed during previous admission
Surgical treatment that did not reach the proposed objective
Lack of diagnosis during previous admission
Healthcare-related infection
Suboptimal medical treatment
Unstable clinical condition at discharge from previous admission
Inadequate use of drugs (includes inadequates dosage and interactions)
Complication of diagnostic test
Nonadherence to treatment allegedly due to lack of information
Diagnostic and/or therapeutic problems should have been treated by primary care services (general physicians, ambulatory, etc.)
Lack of appropriate alternative centres for delivering the care required
Other
***Unavoidable causes***
*Unavoidable recurrence disease*
*Unavoidable progression of disease*
*Process not related to previous episodes*
*Planned readmission* *
*Normal progress of pregnancy*
*Nonadherence to therapeutic recommendations attributable to patient*
*Adverse reaction to drugs (with correct indication and dosage)*
*Acute exacerbation of concomitant process*
*Uncontrollable social problem*
*Other*

Modified by Jiménez-Puente et al. [7].

* Excluding those due to complications from the previous intervention and those indicated for procedures not performed during the previous admission.

A structured record form was used to collect information for each patient self-reported or obtained from medical records on: demographic and socioeconomic characteristics (age, gender, marital status, educational level, working activity), living condition, distance of patient’s home from hospital, pre-admission performance-based measure of basic activities of daily living (BADL) by using the Katz index [14], principal symptom diagnosis and comorbidities at the time of admission using Charlson et al. score [15], detail regarding the admission (day of the week, ward, type, manner of transport, referring authority), length of stay, and discharge destination.

Data were collected also from the first admission charts if the patient had been readmitted. Other data of interest related to the first hospitalization included medical treatment information (adherence, dosage, adverse reactions, drug interactions). Time in days elapsed between the previous admission/index event and the readmission was calculated.

The study protocol was approved by the Ethics Committee of the “Mater Domini” Hospital of Catanzaro (Italy) (Prot. C.E. no.17/10.10.2005).

### Statistical Analysis

Multivariate logistic regression analysis was applied with the aim of identifying the explanatory variables independently related to dichotomous measures of whether or not each patient had a hospital readmission within 30 days of initial admission (Model 1) and, among rehospitalized patients, whether or not the readmission was potentially avoidable (Model 2). The following independent variables were selected and entered into the models: age (≤50 = 1, 51–65 = 2, 66–75 = 3, >75 = 4), gender (male = 1, female = 2), marital status (single/separated/divorced/widowed = 1, married = 2), education (no formal education = 1, primary school = 2, secondary school = 3, high school = 4, college degree = 5), referring authority (general practitioner/emergency department = 1, other = 2), additional person in the household (none = 0, 1 = 1, >1 = 2), distance in kilometers from the patient’s home to the hospital (≤5 = 1, 6–35 = 2, >35 = 3), ward of hospital admission (four categories: General Medicine = 1, Medical Specialties = 2, General Surgery = 3, Surgical Specialties = 4) included as a dummy variable with the General Medicine being the excluded reference category, Katz index of BADL score (0–1 = 0, 2–6 = 1), and Charlson et al. comorbidity score (continuous). In the Model 1, the variables working activity (unemployed/retired = 0, other = 1) and length of hospital stay (continuous) were also included. In the Model 2, the variables length of hospital stay of the previous admission (continuous), type of admission in the readmission episode (scheduled = 1, urgent = 2), and time in days elapsed between discharge from index episode and readmission (continuous) were also included. The significance level for variables entering the models was set at 0.2 and for removing from the model at 0.4. As for logistic regression, results have been expressed as adjusted odds ratios (ORs) with 95% confidence intervals (CIs). These regression analyses assessed the relative strength of the various individual risk factors on readmission status while statistically controlling for other risk factors at the 0.05 statistically significance level. All reported *p* values are two-tailed. All data were analyzed using the Stata version 10.1 statistical software package [16].

## Results

During the study period, 2252 patients were included in the final analysis, equaling a response of 98.4%. The main characteristics of all patients included in the study are presented in Table 2. More than half were males, the mean age was 63.5 years (range 18–99), almost two thirds had attended a primary or secondary school, more than three quarters were admitted to the hospital on a weekday and more than half in medical wards, the mean length of stay was 10.3 days (range 1–80), and the mean Charlson et al. comorbidity score was 0.8 (range 0–12).

**Table 2:** Selected characteristics of the study population.

Characteristic	N	%
**Gender**		
Male	1212	53.8
Female	1040	46.2
**Age group, years**		
≤50	523	23.2
51–65	478	21.2
66–75	567	25.2
>75	684	30.4
Mean±SD	63.5±18.1	
**Education**		
No formal education	413	18.4
Primary school	774	34.4
Secondary school	716	31.9
High school	273	12.1
College degree	72	3.2
**Marital status**		
Married	1530	67.9
Others	722	32.1
**Living condition**		
Alone	177	7.9
1 person	741	32.9
>1 person	1330	59.2
**Working activity**		
Unemployed/retired	1451	64.4
Other	801	35.6
**Patient’s distance home-hospital, km**		
≤5	942	41.8
6–35	757	33.6
>35	553	24.6
**Katz index of BADL** * **score**		
Mean±SD	4±2.6	
**Charlson et al. comorbidity score**		
Mean±SD	0.8±1.2	
**Ward of admission**		
General Medicine	683	30.3
Medical Specialties	570	25.3
General Surgery	376	16.7
Surgical Specialties	623	27.7
**Day of the week of admission**		
Monday-Friday	1784	79.2
Saturday-Sunday	468	20.8
**Type of admission**		
Emergency	412	18.3
Programmed	1840	81.7
**Length of hospital stay, days**		
Mean±SD	10.3±7.9	

* Basic Activities of Daily Living.

The prevalence of the hospitalized patients within 30 days of discharge was 10.2%, and 23 patients had more than one readmission in a period of 30 days. Table 3 presents the distribution of readmission within 30 days of discharge and of potentially avoidable readmission according to various explanatory characteristics. The results of the bivariate statistical analysis showed that several variables were significantly associated with the prevalence of readmission. Indeed, among the socio-demographic characteristics of the patients, a higher prevalence has been observed in older patients (χ^2^ = 15.42, 3 df, *p* = 0.001), in undereducated (χ^2^ = 9.86, 4 df, *p* = 0.043), in unemployed or retired (χ^2^ = 15.98, 1 df, *p*<0.001), in those who lived with one person in the household (χ^2^ = 9.94, 2 df, *p* = 0.007), and if the distance in kilometers from the patient’s home to the hospital was >35 (χ^2^ = 9.11, 2 df, *p* = 0.011). With regard to the functional variables, the readmission prevalence was significantly higher in patients with more comorbidities (t-test = −7.55, 2250 df, *p*<0.001) and in those with a lower Katz index of BADL score (χ^2^ = 8.75, 1 df, *p* = 0.003). Finally, the prevalence was also associated with several hospital characteristics, since a higher value was found in patients admitted on weekday (χ^2^ = 4.68, 1 df, *p* = 0.031), in general surgery wards (χ^2^ = 55.23, 3 df, *p*<0.001), and when the admission was urgent (χ^2^ = 8.22, 1 df, *p* = 0.004). The results of the multivariate logistic regression analysis were not substantially different than those observed in the bivariate analysis and revealed that ward of hospital stay, Charlson et al. comorbidity score, and employment status were significantly associated with the hospital readmission within 30 days of discharge. Indeed, when general medicine wards were chosen as the reference category, the odds of the readmission was significantly higher in general surgery (95% CI = 1.25–2.66, *p* = 0.002) and lower in medical (95% CI = 0.43–0.9, *p* = 0.013) and surgical specialties (95% CI = 0.23–0.59, *p*<0.001). Moreover, the proportion of patients who were readmitted significantly increased regardless of Charlson et al. comorbidity score (95% CI = 1.3–1.87, *p*<0.001) and among the unemployed or retired patients (95% CI = 0.49–0.99, *p* = 0.042) (Model 1 in Table 4).

**Table 3:** Distribution of readmission within 30 days of discharge and of potentially avoidable readmission according to various explanatory variables.

Characteristic		Readmissions within 30 days of discharge		Potentially avoidable readmissions			
		N	%	N		%	
**Gender**							
Male		132	10.9	51		38.6	
Female		97	9.3	49		50.5	
		χ^2^ = 1.49, 1 df, *p* = 0.22		χ^2^ = 3.21, 1 df, *p* = 0.07			
**Age, years**							
≤50		31	5.9	16		51.6	
51–65		48	10	22		45.8	
66–75		72	12.7	30		41.7	
>75		78	11.4	32		41	
		χ^2^ = 15.42, 3 df, *p* = 0.001		χ^2^ = 1.23, 3 df, *p* = 0.74			
**Education**							
No formal education		41	9.9	23		56.1	
Primary school		96	12.4	39		40.6	
Secondary school		70	9.8	26		37.1	
High school		18	6.6	10		55.6	
College degree		4	5.6	2		50	
		χ^2^ = 9.86, 4 df, *p* = 0.043		χ^2^ = 5.25, 4 df, *p* = 0.27			
**Working activity**							
Unemployed/retired		175	12.1	74		42.3	
Other		54	6.7	26		48.2	
		χ^2^ = 15.98, 1 df, *p<*0.001		χ^2^ = 0.58, 1 df, *p = *0.45			
**Living condition**							
Alone		10	5.7	3		30	
1 person		94	12.7	39		41.5	
>1 person		125	9.4	58		46.4	
		χ^2^ = 9.94, 2 df, *p* = 0.007		χ^2^ = 1.32, 2 df, *p* = 0.52			
**Patient’s distance home-hospital, km**							
≤5		97	10.3	40		41.2	
6–35		60	7.9	27		45	
>35		72	13	33		45.8	
		χ^2^ = 9.11, 2 df, *p* = 0.011		χ^2^ = 0.41, 2 df, *p* = 0.81			
**Katz index of BADL** * **score**							
0–1		82	13.2	35		42.7	
2–6		147	9	65		44.2	
		χ^2^ = 8.75, 1 df, *p* = 0.003		χ^2^ = 0.05, 1 df, *p* = 0.82			
**Charlson et al. comorbidity score**							
Mean±SD		1.31±1.74		1.19±1.77			
		t = 7.55, 2250 df, *p<*0.001		t = 0.92, 227 df, *p* = 0.36			
**Ward of admission**							
General Medicine		92	13.5	31		33.7	
Medical Specialties		48	8.4	15		31.3	
General Surgery		64	17	42		65.6	
Surgical Specialties		25	4	12		48	
		χ^2^ = 55.23, 3 df, *p*<0.001		χ^2^ = 19.46, 3 df, *p*<0.001			
**Day of the week of admission**							
Monday-Friday		194	10.9	82		42.3	
Saturday-Sunday		35	7.5	18		51.4	
		χ^2^ = 4.68, 1 df, *p* = 0.031		χ^2^ = 1.01, 1 df, *p* = 0.32			
**Type of admission**							
Urgent		203	11	93		45.8	
Programmed		26	6.3	7		26.9	
		χ^2^ = 8.22, 1 df, *p = *0.004		χ^2^ = 3.34, 1 df, *p = *0.07			
**Time in days elapsed between discharge from index episode and readmission**							
1–10				61		50	
11–30				39		36.5	
					χ^2^ = 4.26, 1 df, *p = *0.039		

* Basic Activities of Daily Living.

**Table 4:** Logistic regression models results.

Variable	OR	SE	95% CI	*p*		
**Model 1. Outcome: Readmissions within 30 days of discharge**						
Log-likelihood = −690.96, χ^2^ = 98.02, *p*<0.001						
Charlson et al. comorbidity index	1.56	0.15	1.3–1.87	<0.001		
Ward of admission						
General Medicine	1.0*	−	−	−		
Medical Specialties	0.62	0.12	0.43–0.9	0.013		
General Surgery	1.83	0.35	1.25–2.66	0.002		
Surgical Specialties	0.37	0.37	0.23–0.59	<0.001		
Working activity	0.7	0.12	0.49–0.99	0.042		
Distance home-hospital, km	1.17	0.1	0.98–1.39	0.08		
Length of hospital stay, days	0.99	0.01	0.97–1.00	0.11		
Gender	0.81	0.12	0.61–1.08	0.16		
**Model 2. Outcome: Potentially avoidable readmissions**						
Log-likelihood = −138.12, χ^2^ = 37.55, *p*<0.001						
Ward of admission						
General Medicine	1.0	−	−	−		
Medical Specialties	0.49	0.22	0.21–1.17	0.11		
General Surgery	3.69	1.38	1.77–7.7	<0.001		
Surgical Specialties	2.21	1.08	0.85–5.76	0.11		
Time in days elapsed between discharge from index episode and readmission	0.96	0.02	0.93–0.99	0.043		
Referring authority	0.42	0.18	0.18–0.99	0.048		
Gender	1.69	0.53	0.91–3.12	0.095		
Day of the week of admission	1.93	0.79	0.86–4.33	0.11		
Education	0.78	0.13	0.56–1.09	0.15		
Marital status	1.47	0.49	0.76–2.81	0.25		

* Reference category.

A total of 100 hospital readmissions (43.7%) were judged as potentially avoidable. In the bivariate analysis, a significant higher number of potentially avoidable readmission was found if the readmission occurred within 10 days after the discharge (χ^2^ = 4.26, 1 df, *p* = 0.039) and if the attending ward in the first hospitalization, was the general surgery (χ^2^ = 19.46, 3 df, *p*<0.001). Of the variables considered in the multivariate logistic regression analysis with potentially avoidable readmission as the dependent variable, the ward of admission, time elapsed between discharge from index episode and readmission, and referring authority were found to be independently associated with the outcome of interest. Patients admitted in general surgery wards being 3.69 times more likely than general medicine to have a potentially avoidable readmission (95% CI = 1.77–7.7, *p*<0.001). The probability of the readmission to be potentially avoidable increased with the decreasing time between discharge and readmission (95% CI = 0.93–0.99, *p* = 0.043). Moreover, the odds of potentially avoidable readmission was significantly higher in patients referred to the hospital by a general practitioner or emergency department physician (95% CI = 0.18–0.99, *p* = 0.048) (Model 2 in Table 4).

Among the reasons cited as casual or contributory to judging the readmission as potentially avoidable were the following: procedures not performed in the first hospitalization (24%), lack of diagnosis in the first hospitalization (19%), unstable clinical condition at discharge from the first hospitalization (17%), and lack of satisfactory primary care (10%). Regarding unavoidable causes, the most frequent reasons listed were: planned readmission, excluding those with complications from the previous intervention and those indicated for procedures not performed during the first hospitalization (25.6%), unavoidable recurrence of disease (21.7%), and acute exacerbation of disease (20.2%).

## Discussion

This investigation measured the prevalence of hospital readmissions within 30 days of discharge and of those readmissions potentially avoidable and this information about such re-hospitalization is vital in determining the appropriateness of resource utilization and in improving the quality of health care.

A comparison of the overall prevalence of hospital readmission reported in the present study with earlier studies suggests that the result observed, 10.2%, was similar to the value of 12.3% which occurred within 30 days in a Department of Internal Medicine in Switzerland [8]. This result was higher than the 5% reported in Internal Medicine departments in Israel [10] but lower than the overall proportion of 30-day unplanned readmission episodes of 16.7% observed among patients admitted to the wards of the Internal Medicine departments of all public hospitals in Hong Kong [17]. The finding of the present study regarding the prevalence of the hospital readmissions at 30 days that were caused by situations potentially avoidable indicated a value of 43.7%. A similar result has been found among medical specialties in the public hospital system in Hong Kong with 40.8% of unplanned readmissions that were judged avoidable [18]. A considerably higher value, 55%, was observed using hospital discharge records accounting in the United States [19]. Moreover, the prevalence is one of the highest encountered in the previous studies. Indeed, in Spain, in a cross-sectional study including patients discharged from a general hospital-care facility, 23.9% of readmissions within one month after discharge were caused by situations potentially avoidable [7]. In Canada, 16% of urgent readmissions of patients aged 18 years or more discharged from medical and surgical services of 11 hospitals after elective or urgent care was considered to be potentially avoidable [20]. Finally, in the already reported study conducted in Switzerland a value of 0.4% of unavoidable readmissions has been reported [8]. It should be pointed out that comparison of the hospital readmissions and of those readmissions potentially avoidable across different geographical areas must be done with caution, since many characteristics may impact hospitalization rates, such as the health care delivery system, the variation in physician practice styles, the lack of consistency in the conditions and the codes used to define them, the methodology and the data analysis used among studies.

The evaluation of the potential role of the different variables that might be associated with the outcomes of interest, according to the multivariate regression analysis, indicated that the ward of hospital readmission, the patient’s health status, the patient’s working activity, the referring authority, and the time elapsed between discharge from previous admission and readmission were the significant determinants that affect the hospital readmission within 30 days of initial admission and whether the readmission was potentially avoidable. Differences existed by ward of hospital readmission, notably because those patients admitted to general surgery wards were more likely to be readmitted and to have a potentially avoidable readmission. The health status, measured in terms of chronic illness according to the Charlson et al. comorbidity score, has also an impact on the prevalence of readmission because the proportion of patients readmitted significantly increased as the value of the comorbidity score increased. It is probable that the nature of chronic diseases makes it difficult to manage patients for extended periods of time, due to deteriorations or exacerbations. In this scenario, primary care plays an important role in the health maintenance of patients with chronic conditions. Readmissions could be related to unsatisfactory access and delivery of primary care, and this accords well with the results of a survey conducted by some of us which indicated that the health of patients with ambulatory care-sensitive conditions, such as hypertension, diabetes, chronic heart failure, chronic obstructive pulmonary disease and asthma deteriorates without access to regular primary care and that this decline contributes to increased hospital use [21]. Moreover, a lower professional category was associated with a higher value of hospital readmission. The current study’s data also showed the role of the time elapsed between discharge from previous hospital admission. Indeed, the frequency of the readmission potentially avoidable increased as the time between discharge and readmission decreased, and more readmissions were judged avoidable when occurring within 0 to 10 days - this result is consistent with the findings of previous studies [18], [22]. It is generally assumed that the shorter the time between discharge and readmission, the more likely it is that the inpatient medical management played a significant role in the readmission [23]. Indeed, the major reasons for avoidable readmissions were lack of diagnosis or procedure not performed during the first hospitalization and unstable clinic condition at discharge. It is well-known that altering the methods of hospital payment (the introduction of the prospective payment system) and the rising demands of hospital services could explain the growth in percentage of patients discharged home leave hospital with an instability. The consequences of premature discharges include elevated hospital readmission prevalence and lower in-patient quality of care [18].

Avoidable readmissions are influenced by factors at the patient, organizational, and environmental levels. Not all of these factors are actually in the hospitals’ control, although the cost of avoidable readmissions will be borne principally by hospitals that can modify their structure and processes. More qualitative information may improve the patient’s ability to manage aspects of his care after discharge, and for the elderly through interviews with the patients, family members, and caregivers about their needs. Education could be supplemented by interventions in which hospital staff are in contact with the patient, with the specific intention of providing support, ease or solve problems after discharge in order to prevent readmissions to the hospital. Moreover, active post-discharge management is one of the reasons that justifies vertical integrated delivery systems that are poised to facilitate transitions between inpatient and outpatient care.

To appreciate the findings of this study, some aspects of the design and measurements need to be discussed as potential threats to the validity of the results. First, the survey was performed in a limited area of Italy, and one could think that this population is not representative of the Italian population as a whole. However, the population of the area surveyed is similar to that of other Italian regions. Second, it is inevitable that cross-sectional investigations do not allow the analysis on the direction of influence between the different variables and the outcomes of interest. However, the aim of the study was to identify the characteristics associated with hospital readmission, in order to target policy focused on reducing further avoidable readmissions. Third, the report of re-hospitalization at the one-month interview might be subject to recall bias, especially if fewer healthy patients are more likely than others to remember with precision the negative events in their lives. Notwithstanding these limitations, the findings presented here are important with respect to risk factors for hospital readmission.

Additional research on intervention or bundle of interventions broadly applicable to acute inpatient populations that aim to reduce potentially avoidable readmissions is strongly needed. In the face of increasingly constrained resources, health care providers are urged to implement evidence-based programs for more cost-effective delivery of health care.
